# Mechanism by Which Tong Xie Yao Fang Heals the Intestinal Mucosa of Rats with Ulcerative Colitis through the Hippo Pathway

**DOI:** 10.1155/2021/5533914

**Published:** 2021-08-30

**Authors:** Wenli You, Zitong Xu, Aiting Di, Penglin Liu, Chengjian Pang, Jianheng Wang, Xiaoyu Li, Yingying Wang, Bin Yu, Xishuang Liu, Gang Zhao

**Affiliations:** ^1^Department of Anorectal, The Affiliated Hospital of Qingdao University, Qingdao, Shandong 266000, China; ^2^Department of Gastroenterology, The Affiliated Hospital of Qingdao University, Qingdao, Shandong 266000, China

## Abstract

**Objective:**

Tong Xie Yao Fang (TXYF) is a classic and effective prescription in traditional Chinese medicine which is used to treat ulcerative colitis (UC). Our study investigated the effect of TXYF on Hippo pathway activation in UC-induced intestinal mucosa injury and explored the possible mechanism.

**Method:**

After ulcerative colitis was successfully induced by trinitrobenzene sulfonic acid (TNBS), 48 Sprague Dawley (SD) rats were randomly divided into a control group, model group, TXYF group, and sulfasalazine group and treated with the corresponding drugs for 28 days. The parameters including body weight, colon length, spleen index, and disease activity index (DAI) and histopathological characteristics were assessed. The myeloperoxidase (MPO) activity and IL-6 level in the colon mucosa were determined with the corresponding commercial kits. The expressions of the Hippo pathway components YAP1, TAZ, P-YAP, and LATS1 were detected in the colon mucosa of each group on different stages by quantitative real-time PCR (qRT-PCR) and western blotting. Immunohistochemical staining was used to evaluate the growth and apoptosis of the colon epithelium.

**Result:**

TXYF significantly improved the weight loss, colonic shortening, DAI, spleen enlargement, and histopathological score of the rats with TNBS-induced UC. TXYF also reduced the MPO activity and expression of IL-6 in the colon mucosa. Furthermore, treatment with TXYF significantly increased YAP1 expression in the early stage (3–7 days) and significantly decreased YAP1 expression in the late stage (14–28 days). In the early stage, TXYF inhibited Hippo pathway activity, which promoted proliferation and regeneration of the intestinal mucosa. In the late stage, the Hippo pathway was activated, thereby inhibiting apoptosis and promoting intestinal mucosal differentiation.

**Conclusion:**

TXYF alleviated the inflammatory response and promoted mucosal healing in rats with UC, which was probably achieved through the Hippo pathway. These results indicated that TXYF was a potential therapy for treating UC.

## 1. Introduction

UC is a chronic inflammatory bowel disease whose aetiology and pathogenesis are unclear. The disease usually affects the mucosa and submucosa of the colon and/or rectum. In recent decades, the incidence rate of UC has notably increased around the world [1, 2]. The predominant symptoms and characteristics of this disease include frequent abdominal pain, bloody diarrhoea, constipation, and fatigue, and these symptoms seriously affect the normal work and lives of patients. Moreover, colorectal cancer is related to long-term chronic intestinal inflammation [3–5]. Thus, UC has become an important global health problem that needs to be considered. However, there is no specific clinical treatment for UC because its mechanism of pathogenesis is unknown. Although conventional medications that contain amino salicylic acid, immunosuppressive agents, and biological agents can alleviate UC, the long-term use of these agents often leads to side effects such as upper gastrointestinal bleeding, vomiting, nausea, headaches, and rashes. In addition, some patients are nonresponsive to such drugs [6]. Therefore, scientists have started to identify alternative and complementary medicines, especially natural medicines with fewer side effects [7, 8].

As a traditional Chinese medicine prescription, TXYF has exhibited obvious therapeutic effects on treating UC, which is why TXYF has been commonly used in China. Recent studies have suggested that TXYF can inhibit inflammation and promote mucosal healing [9, 10], indicating that TXYF plays multiple regulatory roles in the treatment process. However, the therapeutic effect of TXYF has not been scientifically evaluated, and its antiulcer mechanism remains unclear.

It was recently reported that activation of the Hippo pathway in colon stem cells is of great significance for the regeneration and differentiation of colon tissues [11, 12]. The core of this pathway is the kinase cascade reaction. In the early stage of intestinal injury, Hippo pathway activity decreases, which promotes the proliferation of colonic epithelium. In the later stage of intestinal injury, the Hippo pathway is activated. Subsequently, MST1/2 kinase and Sav1 form a phosphorylated complex that activates LATS1/2. Then, LATS1/2 phosphorylation inhibits the transcriptional coactivator YAP1/TAZ. Phosphorylated YAP1 binds to proteins in the cytoplasm and is ubiquitinated and degraded; thus, the abilities of YAP1 to promote proliferation and prevent apoptosis are inhibited [12].

Thus, this study intended to evaluate the therapeutic effect of TXYF on rats with TNBS-induced colitis. In addition, we also revealed the mechanism by which TXYF affects intestinal mucosal healing in UC by altering Hippo pathway activity in the early and late stages of inflammation.

## 2. Materials and Methods

### 2.1. Animals

48 healthy male Sprague Dawley rats (6–8 weeks, 250 ± 10 g) were purchased from Jinan Pengyue Experimental Animal Breeding Co. Ltd. (production licence: SYXK Shandong 20190003). The rats were bred under SPF conditions, an ambient temperature of 23 ± 1°C with 55%–65% humidity, and a 12 : 12-hour light-dark cycle. All the animal care procedures and experiments were approved by the Animal Research Ethics Committee of the Affiliated Hospital of Qingdao University.

### 2.2. UC Rat Model Establishment

Colitis was induced following a previously described method [13, 14]. After being acclimated for 7 days, 48 normal rats were divided into two groups, namely, a control group (*n* = 12) and an experimental group (*n* = 36), according to a random number table. The rats were anaesthetized by inhalation of isoflurane after being fasted for 1 day. The experimental group was administered 100 mg/kg TNBS (Meilunbio, Dalian, China) solution dissolved in isometric 50% ethanol via a thin catheter into the colon. The control group received the same volume of saline via the same route. Then, the rats were held head-down for 1 minute to ensure that the solution was distributed throughout the colon equably. The rats were fed a normal diet after recovering from anaesthesia. On day 3, three rats from the experimental group were stochastically euthanized by an overdose of CO_2_ for the histopathological examination of the colonic tissue to determine whether the UC model was successfully set.

### 2.3. Drug Preparation

All the raw herbal medicines of TXYF were obtained from the Dispensary of Chinese Herb of the Affiliated Hospital of Qingdao University and were identified in accordance with the Pharmacopoeia of People's Republic of China (2015 edition). Rhizoma Atractylodis Macrocephalae (Baizhu), Radix Paeoniae Alba (Shaoyao), Pericarpium Citri Reticulatae (Chenpi), and Radix Saposhnikoviae (Fangfeng) were mixed according to the initial weight ratio (6 : 4 : 3 : 2) of TXYF. Then, the mixture was incubated in 10 volumes of distilled water for 30 minutes and extracted by heating reflux. The herbs were subjected to an initial hard boil and were then simmered for 30 minutes. Next, the hot combined extract was filtered after stirring. Finally, the mixture was condensed to a concentration of 0.78 g/ml, considered the raw herbal medicines, and stored at 4°C. Sulfasalazine enteric-coated tablets (Lot number 09190411, Shanghai Sinepharm Co., Ltd, Shanghai, China) were dissolved in distilled water to 0.03 g/ml.

### 2.4. Animal Groups and Treatments

After successful setting of animal model, we divided the rats into four groups: the control group, model group, TXYF group, and sulfasalazine group. TXYF (10 ml/kg) and sulfasalazine solution were administered by gavage, and the dose was equivalent to a 6.25-fold clinical dose [15]. The control and model groups received the same amount of distilled water via the same route. All the groups received continual intragastric administration once a day after the colitis model was established successfully. All the rats in each group were euthanized with carbon dioxide on days 3, 7, 14, and 28. The colon was removed quickly and cut open lengthwise. The spleen was also removed. The weight and length of the colon tissue and spleen were measured [16]. The harvested tissue was fixed in 4% paraformaldehyde for histopathology and immunohistochemical examination. The other tissue was scraped to collect the mucosa, which was snap-frozen [13] to −80°C until use.

### 2.5. General Assessment of UC Induced by TNBS

To investigate the therapeutic effect of TXYF on TNBS-induced inflammation, we monitored the general conditions of the rats, including weight loss, diarrhoea, and rectal bleeding. The colitis severity and therapeutic effects were assessed with a previously established scoring system [13]. The DAI parameters included the weight ratio, stool consistency, and bleeding, which were evaluated on days 0, 3, 7, 14, and 28 after intragastric administration. The parameters used for the calculation are given in Table 1. The DAI is the sum of the 3 parameters, and it ranged from 0 (unaffected) to 12 (severe colitis). The analysis was carried out independently by three people who were unaware of the experimental conditions.

### 2.6. Histopathological Examination

After being fixed in paraformaldehyde and dehydrated, the colon mucosa tissue was embedded in paraffin. Representative slides were obtained by cutting paraffin sections. Then they were deparaffinized and rehydrated. These slides were also used for immunohistochemical analysis. Some slides were stained with haematoxylin and eosin (H&E staining) and observed by microscopy. As shown in Table 2 [17], the histological score is evaluated under a blinded condition. This index was the sum of the scores ranging from 0 (no damage) to 6 (severe inflammation).

### 2.7. RNA Isolation and RT-qPCR

RT-qPCR was used to assess the mRNA expression levels of YAP1, TAZ, and LATS1. Total RNA was extracted from the colon mucosa using RNA-easy isolation reagent (Vazyme, Nanjing, China). The cDNA was analysed using 2X Universal SYBR Green Fast qPCR mix (ABclonal, Wuhan, China) followed by reverse transcription with an ABscript II cDNA First-Strand Synthesis Kit (ABclonal, Wuhan, China). The RT-qPCR procedure was as follows: 95°C (2 minutes) followed by 40 cycles of 95°C (15 seconds) and 60°C (60 seconds). RT-qPCR was conducted on a real-time qPCR instrument (ABI7500, Applied Biosystems, Foster City, USA). The results were quantified by the 2^−ΔΔCt^ method [18]. GAPDH served as a reference gene. The sequences of the primers used are shown in Table 3.

### 2.8. Western Blot

The protein levels of YAP1, P-YAP, TAZ, LATS1, Claudin-1, and Caspase 3 were evaluated by western blotting. Total protein was extracted from the colon mucosa with RIPA buffer (Beyotime Biotechnology, Shanghai, China) supplemented with protease and phosphatase inhibitors (Epizyme Biotechnology Co., Ltd, Shanghai, China). Then, the proteins were separated and transferred to polyvinylidene difluoride membranes, which were probed with anti-YAP1 monoclonal antibody (1 : 1000), anti-P-YAP antibody (1 : 1000), anti-TAZ monoclonal antibody (1 : 1000), anti-LATS1 monoclonal antibody (1 : 1000), anti-Claudin-1 monoclonal antibody (1 : 1000), and anti-Caspase 3 (1 : 1000, Proteintech Group, Inc., Wuhan, China), respectively. The above primary antibodies were bought from Cell Signaling Technology (USA). Protein bands after overnight incubation with the primary antibodies were incubated with an HRP-labelled antibody (1 : 10000, Epizyme Biotechnology Co., Ltd, Shanghai, China). The results were detected with chemiluminescence detection reagents (Meilun, Dalian, China) following the manufacturer's instructions.

### 2.9. Myeloperoxidase Activity Assay

The activity of myeloperoxidase (MPO), a marker used to evaluate the degree of neutrophil infiltration, in the colonic mucosa was accessed by an MPO assay kit (Nanjing Jiancheng Bioengineering Institute, Nanjing, China) [19]. Colonic homogenate samples were added to a reaction mixture containing H_2_O_2_ and o-anisaniline, and the MPO activity was examined by colorimetric analysis following the instruction.

### 2.10. ELISA

The typical enzyme-linked immunosorbent assay was used to determine the level of IL-6 in the colonic mucosa with a commercial kit (Neobioscience, Shenzhen, China) according to the manufacturer's protocol.

### 2.11. Immunohistochemistry

As mentioned previously, the paraffin slides were used to detect the expressions of YAP1, Ki-67, and Caspase-3 by immunohistochemistry. To retrieve the antigens, the slides were microwaved in sodium citrate buffer (pH 6). The endogenous peroxidase activity of the tissue was blocked by H_2_O_2_ solution, The slides were blocked with 3% bovine serum albumin (BSA) for 30 minutes at room temperature. Then, the tissue slides were incubated with primary antibodies against YAP1 (1 : 400), Ki-67 (1 : 100, Abcam, UK), and Caspase-3 (1 : 200) overnight at 4°C. Subsequently, the intestinal slides were incubated with specific HRP-labelled antibody (1 : 1000) for 50 minutes. The intestinal slides were visualized using the DAB reaction and observed under an optical microscope.

### 2.12. Statistical Analysis

All the statistical results are expressed as the mean ± standard deviation (SD). SPSS 26.0 was used to analyse all the data. Difference between multiple groups with continuous variables was calculated by one-way analysis of variance (ANOVA) with Fisher's least significant difference (LSD) post-test.

## 3. Results

### 3.1. TXYF Significantly Alleviated TNBS-Induced UC

**Figure 1:**
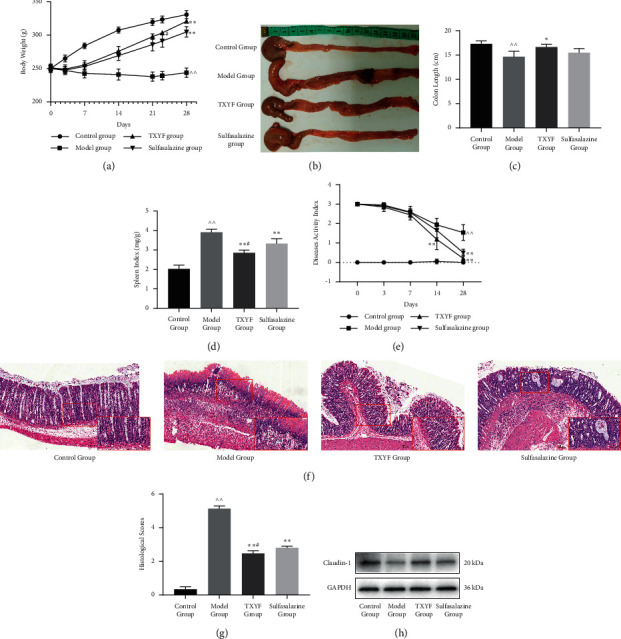
TXYF significantly alleviated UC induced by TNBS. (a) Body weight of every rat was measured per day (*n* = 12 in each group). (b) Colonic length of rats from four groups. (c) Comparison of colon length among four groups (*n* = 3 in each group). (d) Spleen index was calculated by dividing the spleen weight (mg) by the corresponding body weight (g) (*n* = 3 in each group). (e) DAI of four groups (*n* = 12 in each group). (f) The colon was stained with H&E. (g) Histological scores of colon tissue (*n* = 3 in each group). (h) Western blot for Claudin-1 from colon mucosa (*n* = 3 in each group). GAPDH was served as loading control.  ^∧∧^*P* < 0.01 versus control group; ^*∗*^*P* < 0.05 and  ^*∗∗*^*P* < 0.01 versus model group; ^#^*P* < 0.05 and ^##^*P* < 0.01 versus sulfasalazine group.

A rat model of TNBS-induced UC was used to evaluate the treatment of TXYF. The body weight of each experimental rat was approximately the same. During the experimental period, the marked decrease in weight of the rats in the model group compared with that of the rats in the control group was alleviated by TXYF or sulfasalazine treatment. After 22 days, the weight gain of the rats treated with TXYF was significantly higher than that of the rats treated with sulfasalazine (Figure 1(a)). Colon shortening and splenomegaly, which indicated exacerbated colonic inflammation, were reduced by TXYF or sulfasalazine (Figures 1(b)–1(d)). The DAI and histopathological scores are indicators used by physicians to evaluate the activity of UC disease. Compared with the model rats, the DAI of the rats treated with TXYF and sulfasalazine was decreased significantly in the late stage (Figure 1(e)). Furthermore, a significant difference between the TXYF group and sulfasalazine group was obtained in terms of the spleen index and DAI.

Subsequently, we assessed the effect of TXYF on the colon histology of the rats with TNBS-induced colitis. The normal colons had four layers of mucosa with abundant goblet cells, and no sign of obvious inflammatory cell infiltration was observed. However, the colon tissue of the rats from the model group presented a severely damaged mucosal structure, goblet cell loss, inflammatory cell infiltration, mucosal layer destruction, and submucosal oedema. The tissue of the rats with TNBS-induced colitis and treated with TXYF showed reduced morphological changes and significant inflammatory induction with scattered inflammatory cell infiltration. Treatment with sulfasalazine also reduced the level of tissue inflammatory cell infiltration (Figure 1(f)). Compared with the model rats, the rats treated with TXYF exhibited protected intestinal integrity in all the colon layers and significantly decreased histological scores (Figure 1(g)). In addition, TXYF and sulfasalazine reversed the TNBS-mediated decline in Claudin-1 expression (Figure 1(h)).

Taken together, these results revealed that treatment with TXYF suppressed the acute colitis and damage to the mechanical barrier induced by TNBS and promoted mucosal healing.

### 3.2. TXYF Inhibited the Inflammation of Rats with UC

**Figure 2:**
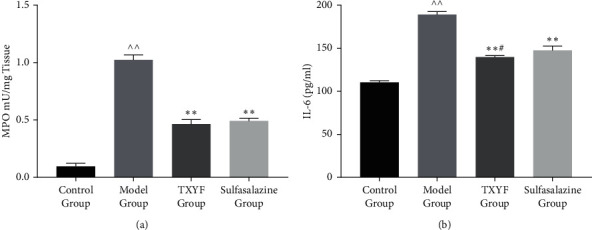
Evaluation of inflammatory response: MPO (a) and IL-6 (b) (*n* = 3 in each group).  ^∧∧^*P* < 0.01 versus control group; ^*∗*^*P* < 0.05 and  ^*∗∗*^*P* < 0.01 versus model group; ^#^*P* < 0.05 and ^##^*P* < 0.01 versus sulfasalazine group.

The pathogenesis of colitis-associated colon cancer is associated with hyperactivation of proinflammatory cytokines [3, 4]. As a marker of neutrophil infiltration, MPO plays an essential role in polymorphonuclear infiltration. As shown in Figure 2(a), treatment with TXYF and sulfasalazine significantly inhibited the MPO activity in the colon tissues. Additionally, we determined the levels of proinflammatory cytokines in the plasma. Compared with that in the rats with UC, IL-6 production in the rats treated with TXYF and sulfasalazine was significantly decreased (Figure 2(b)). These results demonstrated that TXYF could inhibit the inflammatory response in UC.

### 3.3. In the Early Stage of Inflammation (3–7 Days), TXYF Inhibited Hippo Pathway Activation

**Figure 3:**
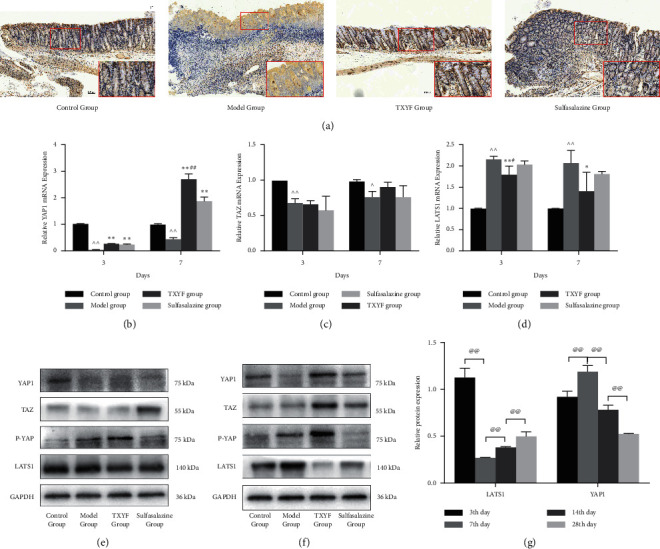
In the early stage of inflammation, TXYF inhibited Hippo pathway activity. (a) The expression of YAP1 from colon tissue was detected by immunohistochemistry on the 7th day. The mRNA levels of YAP1 (b), TAZ (c), and LATS1 (d) from colon mucosa were detected by RT-qPCR analysis on the 7th day. The protein expressions of YAP1, TAZ, P-YAP, and LATS1 from colon mucosa were detected by western blot on the 3rd day (e) and the 7th day (f). (g) The relative protein expression of LATS1 and YAP1 from colon tissue of TXYF group in different stages of colon inflammation. GAPDH was served as internal control (*n* = 3 in each group).  ^∧∧^*P* < 0.01 versus control group; ^*∗*^*P* < 0.05 and  ^*∗∗*^*P* < 0.01 versus model group; ^#^*P* < 0.05 and ^##^*P* < 0.01 versus sulfasalazine group; ^@@^*P* < 0.01 versus the earlier stage.

The Hippo pathway plays an important role in the proliferation, apoptosis, and differentiation of colonic epithelium. In the early stage of injury, the expression of dephosphorylated YAP1 was increased, the Hippo pathway was inhibited, and nucleation was combined with TEADs to induce cell proliferation and inhibit apoptotic gene expression. To investigate the effect of TXYF on the Hippo pathway in the colonic epithelium of rats with TNBS-induced UC during the early stage of inflammation, the expression of YAP1 in the rats treated with or without TXYF was determined by immunohistochemistry. Compared with the rats with UC, the rats treated with TXYF and sulfasalazine exhibited significantly increased expression of YAP1 (Figure 3(a)). Accordingly, the level of LATS1 decreased significantly, while the expression of YAP1 and TAZ increased (Figures 3(b)–3(g)). Additionally, the TXYF group showed an inhibition of Hippo pathway activity comparable to that of the sulfasalazine group (Figures 3(b)–3(f)). These results indicated that TXYF inhibited Hippo pathway activation in the early stage of inflammation and promoted the proliferation and regeneration of colon tissue.

### 3.4. In the Late Stage of Inflammation (14–28 Days), TXYF Increased Hippo Pathway Activity

**Figure 4:**
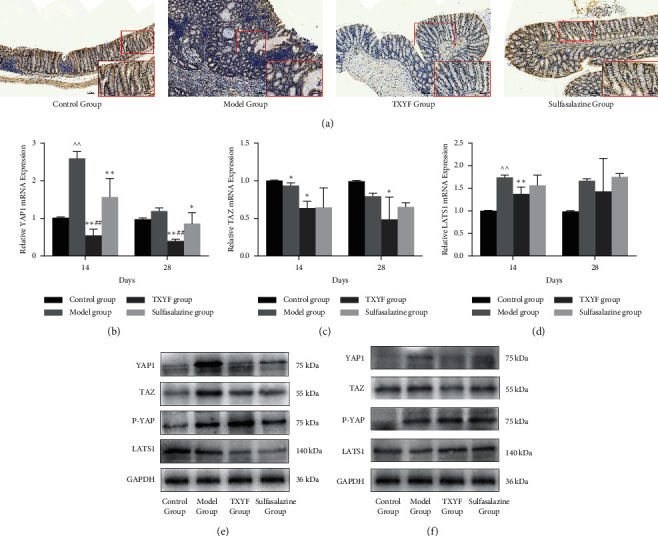
In the late stage of inflammation, TXYF activated Hippo pathway activity. (a) The expression of YAP1 from colon tissues was detected by immunohistochemistry on the 28th day. The mRNA levels of YAP1 (b), TAZ (c), and LATS1 (d) from colon mucosa were detected by RT-qPCR analysis on the 28th day. The protein expressions of YAP1, TAZ, P-YAP, and LATS1 from colon mucosa were detected by western blot on the 14th day and the 28th day.  ^∧∧^*P* < 0.01 versus control group;  ^*∗*^*P* < 0.05 and  ^*∗∗*^*P* < 0.01 versus model group; ^#^*P* < 0.05 and ^##^*P* < 0.01 versus sulfasalazine group.

In the late inflammatory period, the level of YAP1 declined because it was phosphorylated. Hippo pathway activation induces cell differentiation. Compared with that in the model group, the expression of YAP1 in the TXYF group decreased significantly as determined by immunohistochemistry (Figure 4(a)). Accordingly, the level of LATS1 increased significantly, while the expression of YAP1 and TAZ decreased (Figures 4(b)–4(f) and 3(g)). The level of phosphorylated YAP1 increased (Figures 4(b)–4(f)). We found that the level of YAP1 in the TXYF group was significantly higher than that in the model group in the early stage and significantly lower than that in the model group in the late stage. These results showed that TXYF activated the Hippo pathway in the late stage of inflammation, suppressed excessive proliferation, and promoted the restoration of colonic epithelial physiological homeostasis.

### 3.5. Effects of TXYF on Colon Proliferation and Apoptosis

**Figure 5:**
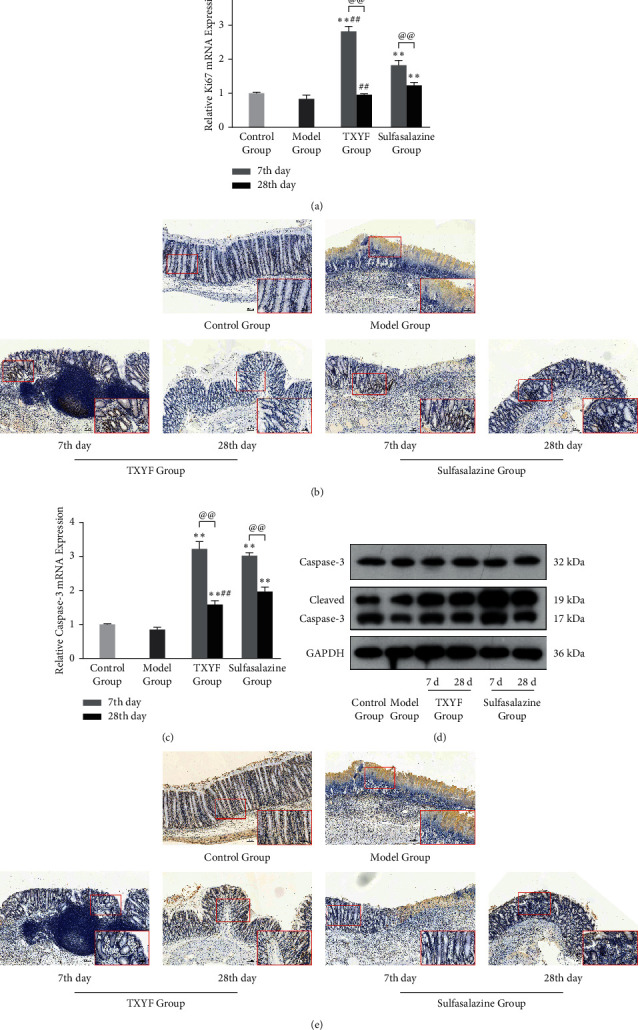
The expression of Ki-67 from colon tissue was detected by RT-qPCR (a) and immunohistochemistry (b). The expression of Caspase-3 from colon tissue was detected by RT-qPCR (c), western blot (d), and immunohistochemistry (e). GAPDH was served as internal control (*n* = 3 in each group).  ^∧∧^*P* < 0.01 versus control group; ^*∗*^*P* < 0.05 and  ^*∗∗*^*P* < 0.01 versus model group; ^#^*P* < 0.05 and ^##^*P* < 0.01 versus sulfasalazine group; ^@@^*P* < 0.01 versus the earlier stage.

The expressions of Ki-67 and Caspase-3 in the colon tissues were determined to evaluate the levels of proliferation and apoptosis. In early stage, TXYF inhibited the Hippo pathway, so the colon cells proliferated significantly, especially at the basement of mucosa. In the late stage of inflammation, TXYF activated the Hippo pathway, inhibiting the proliferation of colonic epithelial cells and reducing the risk of colon cancer transformation (Figures 5(a)-5(b)). Due to severe epithelial necrosis, Caspase-3 could not be detected by immunohistochemistry clearly in the model group, indicating that apoptosis was significant in the early stage of inflammation. After treatment with TXYF or sulfasalazine, the apoptotic activity of colon epithelial cells increased and then decreased in different degrees (Figures 5(c)–5(e)), which may be related to the change of inflammatory response [20].

## 4. Discussion

In our preliminary clinical study, TXYF combined with high-pressure oxygen demonstrated remarkable therapeutic potential for UC (unpublished data), indicating that traditional Chinese medicine is a potential strategy that could be used as a complementary therapy for UC. Therefore, further experimental research is necessary to investigate the anti-UC mechanism of TXYF. Unlike previous studies, we used TNBS to establish an acute UC model in SD rats, which is a classic method for establishing such a model. Compared with the model established using DSS, TNBS results in a longer duration of disease, which can reflect the progression of acute inflammation to chronic inflammation [21]. TXYF is a typical formula used to alleviate abdominal pain and diarrhoea. We used the original ratio of TXYF, which was recorded in Dan Xi Xin Fa (1281 AD), including Rhizoma Atractylodis Macrocephalae (Baizhu), Radix Paeoniae Alba (Shaoyao), Pericarpium Citri Reticulatae (Chenpi), and Radix Saposhnikoviae (Fangfeng). In this study, the improvements in the clinical symptoms and histological conditions were quantified to comprehensively evaluate the therapeutic effect of TXYF on rats with UC (Figure 1). Our results showed that TXYF could improve the weight loss and DAI in rats with UC and ameliorate the pathological injury and inflammatory responses in the colons, which showed the potential of TXYF for treating UC. Therefore, we believe that effective therapy can reduce the risk of intestinal cancer, which is one of the adverse outcomes of UC [3–5]. In addition, TXYF showed an efficacy similar to that of sulfasalazine, which is a classic medicine used for the treatment of UC. Additionally, we have made some new discoveries.

Chronic inflammation is a repetitive process of damage and repair that has been shown to have carcinogenic effects on different organs [22, 23]. Intestinal stem cells (ISCs) perform the difficult task of maintaining a balance between self-renewal and differentiation. Therefore, it is crucial for the treatment of UC to restore the normal proliferation and differentiation functions of colonic epithelial cells. Recent studies indicate that the activity of the Hippo pathway plays a vital role in the regeneration and differentiation of colon tissue [11, 12].

When the Hippo pathway is activated in mammals, the hydrophobic motifs of LATS1/2 can be phosphorylated by MST1/2 and MAP4Ks (mitogen-activated protein kinase, kinase, kinase kinases) to induce their activation [24, 25]. In addition, MST1/2 can phosphorylate and activate SAV1 and MOB1; and the activated SAV1 and MOB1 promote the activity of LATS1/2 [12, 26]. Activated LATS1/2 phosphorylate YAP1 and YAZ, inducing retention of YAP1 and TAZ in cytoplasm by 14-3-3 interaction. Further cytoplasmic phosphorylation and ubiquitination dependent degradation of YAP1 and TAZ were promoted, ultimately inhibiting TEAD-mediated transcription [27–29]. YAP1-TEAD complex can stimulate related genes to promote cell proliferation including CTGF and MYC [30, 31]. Therefore, when the Hippo pathway is activated, phosphorylation of LATS1/2 leads to the degradation of YAP1 and cell proliferation is inhibited. When the Hippo pathway is inhibited, the dephosphorylation expression of YAP1 increases and interact with TEADs to induce cell proliferation. Consistent with previous reports [25, 32–34], LATS1 is a negative regulator of YAP1. As shown in Figure 3(g), TXYF inhibited the Hippo pathway in the early stage of inflammation. The expression of LATS1 was downregulated, while the expression of YAP1 was upregulated. In the late stage of inflammation, the Hippo pathway was activated by TXYF. The expression of LATS1 was gradually upregulated, and the expression of YAP1 was downregulated.

YAP1 is mainly expressed in the cytoplasm of colonic villi and crypt cells [35]. Generally, the activity of YAP1 increases in the initial stage of intestinal repair after injury, promoting the regeneration of intestinal epithelial cells. The decrease in YAP1 activity results in ISCs differentiating and repairing the intestinal tissue in the late stage [36, 37]. Persistent high YAP1 activity in the late stage of inflammation is considered to be a potential risk factor for ulcerative colorectal cancer. Our study showed that the significant decrease in YAP1 in the rats with UC on the third day might be associated with the severe loss and injury of intestinal mucosa. To some extent, both the TXYF and sulfasalazine groups showed improvements in the mucosal injuries caused by TNBS (Figures 1(f)–1(h)). Compared with that in the control group, the Hippo pathway in the model group exhibited dynamic changes of inhibition (upregulation of YAP1 expression) followed by activation (downregulation of YAP1 expression) during the transition from acute to chronic intestinal inflammation. In the model group, the peak of YAP1 activity appeared on the 14th day, which was different from the previously reported peak of YAP1 in DSS-induced colitis on the 2nd–5th day [36, 37]. The difference may be due to the different mechanisms underlying the colonic injury induced by different agents. The peak of YAP1 activity in the TXYF group and the sulfasalazine group appeared on the 7th day, which indicated that TXYF could promote tissue regeneration in the early stage of inflammation. Interestingly, the expression of YAP1 in the TXYF group on the 28th day was obviously lower than those in the other three groups. Moreover, the proliferation of colonic epithelial cells was inhibited.

Notably, the expression of P-YAP was continuously higher on all times during the treatment of TXYF. Inflammation of the colon was still serious on the third day after treatment with TXYF and mucosa had more necrotic tissue. It was possible that a small part of submucosal tissue was scraped during collecting colon tissue, which was not active in the proliferation. Thus, the expression of P-YAP was high. Similar factors might also exist in model group. On the 7th day, the expression of YAP1 of TXYF group in the basement of mucosa increased, indicating active proliferation as shown by immunohistochemistry (Figure 3(a)). The expression of YAP1 in villus was downregulated compared with that in the basement, indicating low proliferation activity of cells and tendency to differentiate. The authors suggest that TXYF may not only promote the proliferation of colon cells in the early stage of inflammation but also promote the differentiation of some cells, so P-YAP also expresses in the early stage of inflammation. Compared with the model group on the 14th day, the simultaneous expression peaks of YAP1 and P-YAP appeared, indicating that TXYF had the potential to advance the peak of proliferation and differentiation. The expression of P-YAP was high on the 14th day after treating with TXYF, while the expression of YAP was significantly downregulated. The state of excessive proliferation was inhibited by TXYF in the late stage of inflammation when differentiation of colon epithelium played an important role. On the 28th day, the expression of P-YAP in TXYF group was higher than those in model group and sulfasalazine group, indicating that it was more beneficial to promoting differentiation of colon tissue in the late stage of inflammation.

The multicomponent and multitarget effects of TXYF may play a bidirectional role in regulating the Hippo pathway at different stages of disease, thus restoring the regeneration of dysfunctional intestinal epithelial cells and homeostasis in vivo.

## 5. Conclusions

TXYF alleviated the inflammatory response and promoted mucosal healing in rats with UC, which was probably achieved through the Hippo pathway. Our study provides new evidence that reveals, for the first time, the potential of TXYF, including its significant effects in the treatment of UC and the reduction of cancer risk.

## Figures and Tables

**Table 1:** Evaluation of disease activity index (DAI).

Score (s)	Weight loss (%)	Stool consistency	Bleeding
0	None	Normal	Normal
1	1–5	—	—
2	5–10	Loose stools	Hemoccult positive
3	10–15	—	—
4	More than 15	Watery diarrhoea	Gross bleeding

**Table 2:** Evaluation of histological scores.

Score (s)	Inflammatory cells	Tissue damage
0	Occasional inflammatory cells in the lamina propria	No mucosal damage
1	Increased numbers of inflammatory cells in the lamina propria	Discrete lymphoepithelial lesions
2	Confluence of inflammatory cells, extending into the submucosa	Surface mucosal erosion or focal ulceration
3	Transmural extension of the infiltrate	Extensive mucosal damage and extension into deeper structures of the bowel wall

**Table 3:** The sequences of the RT-qPCR primers.

Gene	Forward primer (5′-3′)	Reverse primer (5′-3′)
YAP1	GTCAGACCGTCAGAGCGGG	AGGCCACTGTCTGTGCTCTC
TAZ	TCCAGCTCGTCAGTTCGGGA	TTCGAGGTCCGTGTCGAGGT
LATS1	CAGAGTTACTCCCCGCAGGC	TGGGCATCTTGAGATAATCCAACCC
Ki-67	CTTTATGGCTGCTGGGTGCT	GAGGTTGAAGCCGGACACAC
Caspase-3	GAGGTTGAAGCCGGACACAC	TCCAGAGTCCATCGACTTGCTT
GAPDH	CAGCCGCATCTTCTTGTGC	GGTAACCAGGCGTCCGATA

## Data Availability

The data used to support the study are available from the lead author upon request.
